# 3-Ethyl 2-methyl 8-bromo-2-phenyl-1,2,3,3a,4,9b-hexa­hydro­chromeno[4,3-*b*]pyrrole-2,3-dicarboxyl­ate

**DOI:** 10.1107/S1600536810044491

**Published:** 2010-11-06

**Authors:** Long He

**Affiliations:** a College of Chemistry and Chemical Engineering, China West Normal University, Nanchong 637002, People’s Republic of China

## Abstract

The title compound, C_22_H_22_BrNO_5_, was synthesized by the intra­molecular cyclo­addition reaction of (*E*)-ethyl 4-(4-bromo-2-formyl­phen­oxy)but-2-enoate and methyl 2-amino-2-phenyl­acetate. The pyrrolidine and 3,4-dihydro-2*H*-pyran rings exhibit envelope conformations. The two benzene rings are twisted to each other at a dihedral angle of 59.36 (18)°. The eth­oxy group of the ester unit is disordered over two sites with an occupancy ratio of 0.503 (11):0.497 (11). Weak inter­molecular C—H⋯O hydrogen bonding is present in the crystal structure.

## Related literature

For the biological activity of pyrrolidine derivatives, see: Coldham & Hufton (2005 ▶); Grigg (1995 ▶); Kravchenko *et al.* (2005 ▶); Nair & Suja (2007 ▶); Pandey *et al.* (2006 ▶); Sardina & Rapoport (1996 ▶); Witherup *et al.* (1995 ▶). For a related structure, see: Yu *et al.* (2007 ▶).
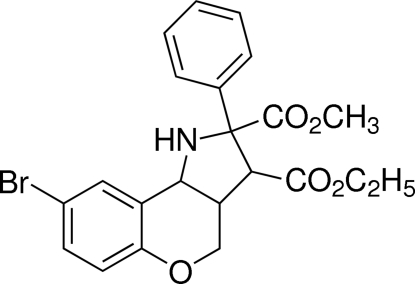


## Experimental

### 

#### Crystal data

C_22_H_22_BrNO_5_
*M* *_r_* = 460.32
Monoclinic, 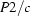

*a* = 11.1046 (8) Å
*b* = 11.1633 (6) Å
*c* = 17.9779 (9) Å
β = 107.856 (6)°
*V* = 2121.3 (2) Å^3^
*Z* = 4
Mo *K*α radiation
μ = 1.97 mm^−1^
*T* = 293 K
0.50 × 0.42 × 0.38 mm

#### Data collection

Oxford diffraction Gemini S Ultra diffractometer
Absorption correction: multi-scan (*CrysAlis PRO*; Oxford Diffraction, 2009 ▶) *T*_min_ = 0.439, *T*_max_ = 0.521
10601 measured reflections
3609 independent reflections
2015 reflections with *I* > 2σ(*I*)
*R*_int_ = 0.027

#### Refinement

*R*[*F* ^2^ > 2σ(*F* ^2^)] = 0.047
*wR*(*F* ^2^) = 0.137
*S* = 1.06
3609 reflections
293 parameters
47 restraints
H-atom parameters constrained
Δρ_max_ = 0.53 e Å^−3^
Δρ_min_ = −0.36 e Å^−3^

### 

Data collection: *CrysAlis CCD* (Oxford Diffraction, 2008 ▶); cell refinement: *CrysAlis RED* (Oxford Diffraction, 2008 ▶); data reduction: *CrysAlis RED*; program(s) used to solve structure: *SHELXS97* (Sheldrick, 2008 ▶); program(s) used to refine structure: *SHELXL97* (Sheldrick, 2008 ▶); molecular graphics: *ORTEP-3* (Farrugia, 1997 ▶); software used to prepare material for publication: *SHELXL97*.

## Supplementary Material

Crystal structure: contains datablocks global, I. DOI: 10.1107/S1600536810044491/xu5063sup1.cif

Structure factors: contains datablocks I. DOI: 10.1107/S1600536810044491/xu5063Isup2.hkl

Additional supplementary materials:  crystallographic information; 3D view; checkCIF report

## Figures and Tables

**Table 1:** Hydrogen-bond geometry (Å, °)

*D*—H⋯*A*	*D*—H	H⋯*A*	*D*⋯*A*	*D*—H⋯*A*
C8—H8⋯O5^i^	0.98	2.37	3.317 (5)	163 (1)

Symmetry code: (i) 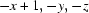.

**Fig. 1.**
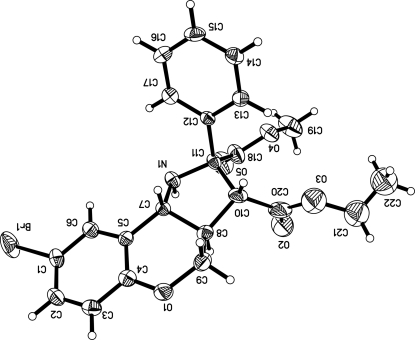
The molecular structure of (I) with 30% probability displacement ellipsoids (arbitrary spheres for H atoms).

## supplementary crystallographic information

### Comment

Pyrrolidine containing compounds are an important class of heterocyclic compounds with wide spread applications to the synthesis of biologically active compounds and natural products. (Coldham *et al.*, 2005; Grigg *et al.*, 1995; Kravchenko *et al.*, 2005; Nair *et al.*, 2007; Pandey *et al.*, 2006; Sardina *et al.*, 1996; Witherup *et al.* 1995). Its crystal structure is reported here.

The molecular structure of (I) is shown in Fig. 1. Bond lengths and angles in (I) are normal. The pyrrolidine ring possesses an envelope conformation. The dihedral angle between the C1—C6 and C12—C17 benzene planes is 59.38 (8)°. The crystal packing is stabilized by C—H···0 hydrogen bonding (Table 1).

### Experimental

(*E*)-Ethyl 4-(4-bromo-2-formylphenoxy)but-2-enoate (0.0374 g, 0.12 mmol) and phosphenous acid (5 mg, 0.01 mmol) were added to a solution of methyl 2-amino-2-phenylacetate (0.016 g, 0.1 mmol) in dichloromethane (1 ml). After the mixture had been stirred at 298 K for 24 h, the reaction was quenched with a saturated solution of sodium bicarbonate (5 ml). The mixture was extracted with ethyl acetate, evaporated and separated by flash chromatograghy. A colourless powder was obtained. Single crystals suitable for X-ray diffraction were obtained by slow evaporation of an ethanol solution.

### Refinement

H atom on N atom was located in a difference Fourier map and refined isotropically. Other H atoms were placed in calculated positions with C—H = 0.93–0.98 Å, and refined using a riding model, with *U*_iso_(H) =1.2*U*_eq_(C).

### Crystal data

**Table:**

C_22_H_22_BrNO_5_	*F*(000) = 944
*M**_r_* = 460.32	*D*_x_ = 1.441 Mg m^−^^3^
Monoclinic, *P*2/*c*	Mo *K*α radiation, λ = 0.71073 Å
Hall symbol: -P 2yc	Cell parameters from 4173 reflections
*a* = 11.1046 (8) Å	θ = 3.0–29.1°
*b* = 11.1633 (6) Å	µ = 1.97 mm^−^^1^
*c* = 17.9779 (9) Å	*T* = 293 K
β = 107.856 (6)°	Block, colorless
*V* = 2121.3 (2) Å^3^	0.50 × 0.42 × 0.38 mm
*Z* = 4	

### Data collection

**Table:**

Oxford diffraction Gemini S Ultra diffractometer	3609 independent reflections
Radiation source: Enhance (Mo) X-ray Source	2015 reflections with *I* > 2σ(*I*)
graphite	*R*_int_ = 0.027
Detector resolution: 15.9149 pixels mm^-1^	θ_max_ = 25.0°, θ_min_ = 3.0°
ω scans	*h* = −10→13
Absorption correction: multi-scan (*CrysAlis PRO*; Oxford Diffraction, 2009)	*k* = −13→13
*T*_min_ = 0.439, *T*_max_ = 0.521	*l* = −21→14
10601 measured reflections	

### Refinement

**Table:**

Refinement on *F*^2^	Secondary atom site location: difference Fourier map
Least-squares matrix: full	Hydrogen site location: inferred from neighbouring sites
*R*[*F*^2^ > 2σ(*F*^2^)] = 0.047	H-atom parameters constrained
*wR*(*F*^2^) = 0.137	*w* = 1/[σ^2^(*F*_o_^2^) + (0.070*P*)^2^] where *P* = (*F*_o_^2^ + 2*F*_c_^2^)/3
*S* = 1.06	(Δ/σ)_max_ < 0.001
3609 reflections	Δρ_max_ = 0.53 e Å^−^^3^
293 parameters	Δρ_min_ = −0.36 e Å^−^^3^
47 restraints	Extinction correction: *SHELXL97* (Sheldrick, 2008), Fc^*^=kFc[1+0.001xFc^2^λ^3^/sin(2θ)]^-1/4^
Primary atom site location: structure-invariant direct methods	Extinction coefficient: 0.0095 (11)

### Special details

**Table:**

Geometry. All e.s.d.'s (except the e.s.d. in the dihedral angle between two l.s. planes) are estimated using the full covariance matrix. The cell e.s.d.'s are taken into account individually in the estimation of e.s.d.'s in distances, angles and torsion angles; correlations between e.s.d.'s in cell parameters are only used when they are defined by crystal symmetry. An approximate (isotropic) treatment of cell e.s.d.'s is used for estimating e.s.d.'s involving l.s. planes.
Refinement. Refinement of *F*^2^ against ALL reflections. The weighted *R*-factor *wR* and goodness of fit *S* are based on *F*^2^, conventional *R*-factors *R* are based on *F*, with *F* set to zero for negative *F*^2^. The threshold expression of *F*^2^ > σ(*F*^2^) is used only for calculating *R*-factors(gt) *etc*. and is not relevant to the choice of reflections for refinement. *R*-factors based on *F*^2^ are statistically about twice as large as those based on *F*, and *R*- factors based on ALL data will be even larger.

### Fractional atomic coordinates and isotropic or equivalent isotropic displacement parameters (Å^2^)

**Table:**

	*x*	*y*	*z*	*U*_iso_*/*U*_eq_	Occ. (<1)
Br1	0.72424 (6)	0.38095 (6)	0.29704 (3)	0.1076 (4)	
O1	0.6457 (3)	0.3997 (2)	−0.04947 (15)	0.0595 (8)	
O2	0.4138 (4)	0.0088 (4)	−0.1551 (2)	0.1059 (12)	
O4	0.1640 (3)	−0.0047 (2)	−0.11722 (17)	0.0635 (8)	
O5	0.3115 (4)	−0.0409 (3)	−0.0070 (2)	0.1175 (15)	
N1	0.3815 (3)	0.1909 (3)	0.01891 (15)	0.0449 (8)	
H1	0.4243	0.1231	0.0420	0.054*	
C1	0.6979 (4)	0.3818 (3)	0.1873 (2)	0.0533 (11)	
C2	0.7856 (4)	0.4379 (3)	0.1590 (2)	0.0551 (11)	
H2	0.8577	0.4723	0.1932	0.066*	
C3	0.7649 (4)	0.4422 (3)	0.0789 (2)	0.0511 (10)	
H3	0.8230	0.4803	0.0590	0.061*	
C4	0.6575 (4)	0.3898 (3)	0.0286 (2)	0.0448 (10)	
C5	0.5689 (3)	0.3342 (3)	0.0584 (2)	0.0410 (9)	
C6	0.5906 (4)	0.3296 (3)	0.1377 (2)	0.0473 (10)	
H6	0.5332	0.2913	0.1579	0.057*	
C7	0.4515 (3)	0.2897 (3)	−0.00127 (18)	0.0367 (9)	
H7	0.3931	0.3577	−0.0154	0.044*	
C8	0.4777 (3)	0.2507 (3)	−0.07426 (19)	0.0442 (9)	
H8	0.5386	0.1845	−0.0617	0.053*	
C9	0.5333 (4)	0.3526 (3)	−0.1078 (2)	0.0553 (11)	
H9B	0.5556	0.3247	−0.1530	0.066*	
H9A	0.4709	0.4158	−0.1248	0.066*	
C10	0.3478 (3)	0.2018 (3)	−0.1234 (2)	0.0475 (10)	
H10	0.2977	0.2678	−0.1535	0.057*	
C11	0.2866 (3)	0.1615 (3)	−0.0566 (2)	0.0436 (10)	
C12	0.1641 (3)	0.2301 (3)	−0.0647 (2)	0.0387 (9)	
C13	0.0758 (3)	0.2521 (3)	−0.1367 (2)	0.0466 (10)	
H13	0.0913	0.2254	−0.1819	0.056*	
C14	−0.0350 (4)	0.3130 (4)	−0.1424 (2)	0.0563 (11)	
H14	−0.0926	0.3285	−0.1912	0.068*	
C15	−0.0600 (4)	0.3508 (3)	−0.0757 (3)	0.0593 (11)	
H15	−0.1355	0.3900	−0.0792	0.071*	
C16	0.0266 (4)	0.3306 (3)	−0.0044 (2)	0.0570 (11)	
H16	0.0108	0.3581	0.0406	0.068*	
C17	0.1375 (4)	0.2696 (3)	0.0017 (2)	0.0457 (9)	
H17	0.1948	0.2549	0.0507	0.055*	
C18	0.2588 (4)	0.0276 (4)	−0.0581 (2)	0.0618 (12)	
C19	0.1333 (5)	−0.1312 (3)	−0.1246 (3)	0.0905 (17)	
H19B	0.0627	−0.1443	−0.1706	0.136*	
H19A	0.1119	−0.1576	−0.0794	0.136*	
H19C	0.2050	−0.1757	−0.1287	0.136*	
C20	0.3562 (4)	0.1050 (5)	−0.1778 (3)	0.0756 (15)	
O3A	0.2757 (11)	0.1020 (10)	−0.2478 (4)	0.077 (4)	0.503 (11)
C21A	0.2932 (13)	−0.0032 (13)	−0.2963 (6)	0.085 (4)	0.503 (11)
H21A	0.3737	−0.0010	−0.3069	0.102*	0.503 (11)
H21D	0.2828	−0.0796	−0.2733	0.102*	0.503 (11)
C22A	0.1844 (14)	0.0266 (14)	−0.3665 (7)	0.114 (5)	0.503 (11)
H22A	0.1644	−0.0415	−0.4007	0.137*	0.503 (11)
H22B	0.2065	0.0931	−0.3935	0.137*	0.503 (11)
H22C	0.1122	0.0472	−0.3504	0.137*	0.503 (11)
O3B	0.3037 (11)	0.1466 (10)	−0.2494 (4)	0.076 (4)	0.497 (11)
C21B	0.2937 (12)	0.0691 (13)	−0.3194 (7)	0.087 (4)	0.497 (11)
H21B	0.2733	0.1182	−0.3662	0.104*	0.497 (11)
H21C	0.3740	0.0299	−0.3135	0.104*	0.497 (11)
C22B	0.1929 (17)	−0.0226 (13)	−0.3272 (11)	0.119 (5)	0.497 (11)
H22D	0.2015	−0.0845	−0.3624	0.143*	0.497 (11)
H22E	0.1114	0.0145	−0.3474	0.143*	0.497 (11)
H22F	0.2011	−0.0568	−0.2769	0.143*	0.497 (11)

### Atomic displacement parameters (Å^2^)

**Table:**

	*U*^11^	*U*^22^	*U*^33^	*U*^12^	*U*^13^	*U*^23^
Br1	0.1153 (6)	0.1448 (6)	0.0475 (3)	−0.0492 (4)	0.0024 (3)	−0.0107 (3)
O1	0.0545 (19)	0.0725 (19)	0.0533 (18)	−0.0083 (14)	0.0191 (14)	0.0055 (13)
O2	0.088 (3)	0.098 (3)	0.127 (3)	0.007 (2)	0.026 (2)	−0.052 (2)
O4	0.0518 (19)	0.0337 (16)	0.089 (2)	0.0030 (13)	−0.0016 (15)	−0.0052 (13)
O5	0.119 (3)	0.059 (2)	0.123 (3)	0.009 (2)	−0.039 (2)	0.015 (2)
N1	0.0409 (19)	0.0413 (18)	0.0432 (17)	0.0029 (15)	−0.0008 (14)	0.0036 (14)
C1	0.058 (3)	0.051 (2)	0.042 (2)	−0.004 (2)	0.003 (2)	−0.0067 (18)
C2	0.047 (3)	0.041 (2)	0.068 (3)	−0.003 (2)	0.005 (2)	−0.008 (2)
C3	0.038 (3)	0.043 (2)	0.074 (3)	0.0021 (18)	0.018 (2)	0.004 (2)
C4	0.044 (3)	0.035 (2)	0.055 (3)	0.0114 (19)	0.014 (2)	0.0039 (18)
C5	0.040 (2)	0.030 (2)	0.052 (2)	0.0079 (17)	0.0133 (19)	−0.0003 (17)
C6	0.049 (3)	0.045 (2)	0.047 (2)	−0.0046 (19)	0.0130 (19)	0.0014 (18)
C7	0.037 (2)	0.034 (2)	0.0370 (19)	0.0028 (17)	0.0082 (17)	0.0018 (15)
C8	0.036 (2)	0.042 (2)	0.054 (2)	0.0083 (17)	0.0133 (18)	−0.0024 (18)
C9	0.058 (3)	0.063 (3)	0.047 (2)	0.006 (2)	0.020 (2)	−0.0011 (19)
C10	0.043 (2)	0.056 (3)	0.041 (2)	0.0141 (19)	0.0085 (18)	−0.0065 (18)
C11	0.036 (2)	0.038 (2)	0.050 (2)	0.0032 (17)	0.0018 (17)	−0.0037 (17)
C12	0.039 (2)	0.0276 (19)	0.046 (2)	−0.0025 (16)	0.0078 (18)	−0.0004 (16)
C13	0.044 (3)	0.050 (2)	0.043 (2)	0.0046 (19)	0.0091 (18)	−0.0048 (17)
C14	0.047 (3)	0.059 (3)	0.056 (3)	0.009 (2)	0.005 (2)	−0.006 (2)
C15	0.044 (3)	0.052 (3)	0.085 (3)	0.010 (2)	0.024 (2)	0.006 (2)
C16	0.062 (3)	0.055 (3)	0.061 (3)	0.004 (2)	0.029 (2)	−0.003 (2)
C17	0.045 (3)	0.044 (2)	0.049 (2)	−0.0008 (19)	0.0160 (19)	0.0012 (17)
C18	0.057 (3)	0.043 (3)	0.067 (3)	0.013 (2)	−0.007 (2)	0.002 (2)
C19	0.080 (4)	0.038 (3)	0.134 (5)	−0.008 (2)	0.004 (3)	−0.009 (2)
C20	0.046 (3)	0.101 (4)	0.076 (4)	0.014 (3)	0.012 (3)	−0.034 (3)
O3A	0.076 (7)	0.074 (6)	0.064 (5)	−0.009 (5)	−0.006 (4)	−0.048 (4)
C21A	0.096 (6)	0.100 (7)	0.060 (6)	−0.012 (6)	0.024 (5)	−0.031 (5)
C22A	0.128 (8)	0.095 (8)	0.103 (8)	−0.006 (6)	0.011 (6)	−0.028 (6)
O3B	0.062 (6)	0.103 (9)	0.058 (5)	−0.024 (6)	0.012 (4)	−0.057 (5)
C21B	0.088 (6)	0.106 (7)	0.065 (6)	−0.004 (6)	0.022 (5)	−0.023 (6)
C22B	0.120 (8)	0.120 (9)	0.116 (9)	−0.011 (7)	0.034 (7)	−0.036 (7)

### Geometric parameters (Å, °)

**Table:**

Br1—C1	1.904 (4)	C11—C12	1.530 (5)
O1—C4	1.374 (4)	C12—C13	1.384 (4)
O1—C9	1.460 (5)	C12—C17	1.387 (5)
O2—C20	1.253 (6)	C13—C14	1.382 (5)
O4—C18	1.297 (4)	C13—H13	0.9300
O4—C19	1.449 (4)	C14—C15	1.377 (5)
O5—C18	1.201 (5)	C14—H14	0.9300
N1—C7	1.458 (4)	C15—C16	1.365 (6)
N1—C11	1.478 (4)	C15—H15	0.9300
N1—H1	0.9221	C16—C17	1.382 (5)
C1—C6	1.379 (5)	C16—H16	0.9300
C1—C2	1.379 (6)	C17—H17	0.9300
C2—C3	1.387 (5)	C19—H19B	0.9600
C2—H2	0.9300	C19—H19A	0.9600
C3—C4	1.387 (5)	C19—H19C	0.9600
C3—H3	0.9300	C20—O3A	1.301 (8)
C4—C5	1.400 (5)	C20—O3B	1.323 (8)
C5—C6	1.372 (5)	O3A—C21A	1.510 (9)
C5—C7	1.496 (5)	C21A—C22A	1.493 (10)
C6—H6	0.9300	C21A—H21A	0.9700
C7—C8	1.493 (5)	C21A—H21D	0.9700
C7—H7	0.9800	C22A—H22A	0.9600
C8—C9	1.505 (5)	C22A—H22B	0.9600
C8—C10	1.542 (5)	C22A—H22C	0.9600
C8—H8	0.9800	O3B—C21B	1.503 (9)
C9—H9B	0.9700	C21B—C22B	1.491 (10)
C9—H9A	0.9700	C21B—H21B	0.9700
C10—C20	1.480 (6)	C21B—H21C	0.9700
C10—C11	1.614 (5)	C22B—H22D	0.9600
C10—H10	0.9800	C22B—H22E	0.9600
C11—C18	1.524 (5)	C22B—H22F	0.9600
			
C4—O1—C9	119.8 (3)	C13—C12—C17	118.1 (3)
C18—O4—C19	117.1 (3)	C13—C12—C11	122.2 (3)
C7—N1—C11	102.9 (3)	C17—C12—C11	119.7 (3)
C7—N1—H1	119.4	C14—C13—C12	121.1 (3)
C11—N1—H1	110.8	C14—C13—H13	119.5
C6—C1—C2	121.4 (4)	C12—C13—H13	119.5
C6—C1—Br1	119.7 (3)	C15—C14—C13	119.9 (4)
C2—C1—Br1	118.8 (3)	C15—C14—H14	120.0
C1—C2—C3	119.2 (4)	C13—C14—H14	120.0
C1—C2—H2	120.4	C16—C15—C14	119.7 (4)
C3—C2—H2	120.4	C16—C15—H15	120.2
C4—C3—C2	119.8 (4)	C14—C15—H15	120.2
C4—C3—H3	120.1	C15—C16—C17	120.7 (4)
C2—C3—H3	120.1	C15—C16—H16	119.7
O1—C4—C3	115.2 (3)	C17—C16—H16	119.7
O1—C4—C5	124.6 (3)	C16—C17—C12	120.5 (3)
C3—C4—C5	120.2 (4)	C16—C17—H17	119.7
C6—C5—C4	119.6 (3)	C12—C17—H17	119.7
C6—C5—C7	124.7 (3)	O5—C18—O4	122.2 (4)
C4—C5—C7	115.7 (3)	O5—C18—C11	124.2 (4)
C5—C6—C1	119.8 (4)	O4—C18—C11	113.4 (3)
C5—C6—H6	120.1	O4—C19—H19B	109.5
C1—C6—H6	120.1	O4—C19—H19A	109.5
N1—C7—C8	105.1 (3)	H19B—C19—H19A	109.5
N1—C7—C5	119.1 (3)	O4—C19—H19C	109.5
C8—C7—C5	111.4 (3)	H19B—C19—H19C	109.5
N1—C7—H7	106.8	H19A—C19—H19C	109.5
C8—C7—H7	106.8	O2—C20—O3A	115.1 (6)
C5—C7—H7	106.8	O2—C20—O3B	130.2 (6)
C7—C8—C9	110.3 (3)	O2—C20—C10	122.7 (5)
C7—C8—C10	101.9 (3)	O3A—C20—C10	119.7 (6)
C9—C8—C10	117.7 (3)	O3B—C20—C10	106.8 (6)
C7—C8—H8	108.9	C20—O3A—C21A	114.0 (8)
C9—C8—H8	108.9	C22A—C21A—O3A	95.8 (8)
C10—C8—H8	108.9	C22A—C21A—H21A	112.6
O1—C9—C8	110.5 (3)	O3A—C21A—H21A	112.6
O1—C9—H9B	109.6	C22A—C21A—H21D	112.6
C8—C9—H9B	109.6	O3A—C21A—H21D	112.6
O1—C9—H9A	109.6	H21A—C21A—H21D	110.1
C8—C9—H9A	109.6	C20—O3B—C21B	120.7 (9)
H9B—C9—H9A	108.1	C22B—C21B—O3B	109.6 (10)
C20—C10—C8	113.6 (3)	C22B—C21B—H21B	109.8
C20—C10—C11	114.5 (4)	O3B—C21B—H21B	109.8
C8—C10—C11	101.9 (3)	C22B—C21B—H21C	109.8
C20—C10—H10	108.9	O3B—C21B—H21C	109.8
C8—C10—H10	108.9	H21B—C21B—H21C	108.2
C11—C10—H10	108.9	C21B—C22B—H22D	109.5
N1—C11—C18	108.5 (3)	C21B—C22B—H22E	109.5
N1—C11—C12	109.8 (3)	H22D—C22B—H22E	109.5
C18—C11—C12	108.7 (3)	C21B—C22B—H22F	109.5
N1—C11—C10	106.1 (3)	H22D—C22B—H22F	109.5
C18—C11—C10	112.9 (3)	H22E—C22B—H22F	109.5
C12—C11—C10	110.8 (3)		
			
C6—C1—C2—C3	0.3 (6)	C20—C10—C11—C12	−117.3 (3)
Br1—C1—C2—C3	−177.9 (3)	C8—C10—C11—C12	119.7 (3)
C1—C2—C3—C4	−0.4 (6)	N1—C11—C12—C13	158.3 (3)
C9—O1—C4—C3	−177.2 (3)	C18—C11—C12—C13	−83.2 (4)
C9—O1—C4—C5	1.3 (5)	C10—C11—C12—C13	41.4 (4)
C2—C3—C4—O1	179.6 (3)	N1—C11—C12—C17	−23.5 (4)
C2—C3—C4—C5	1.1 (5)	C18—C11—C12—C17	95.1 (4)
O1—C4—C5—C6	−180.0 (3)	C10—C11—C12—C17	−140.4 (3)
C3—C4—C5—C6	−1.6 (5)	C17—C12—C13—C14	0.9 (5)
O1—C4—C5—C7	−3.3 (5)	C11—C12—C13—C14	179.2 (3)
C3—C4—C5—C7	175.1 (3)	C12—C13—C14—C15	−1.3 (6)
C4—C5—C6—C1	1.5 (5)	C13—C14—C15—C16	1.8 (6)
C7—C5—C6—C1	−174.9 (3)	C14—C15—C16—C17	−1.8 (6)
C2—C1—C6—C5	−0.9 (6)	C15—C16—C17—C12	1.4 (6)
Br1—C1—C6—C5	177.4 (3)	C13—C12—C17—C16	−1.0 (5)
C11—N1—C7—C8	−45.9 (3)	C11—C12—C17—C16	−179.3 (3)
C11—N1—C7—C5	−171.6 (3)	C19—O4—C18—O5	−7.6 (7)
C6—C5—C7—N1	−29.2 (5)	C19—O4—C18—C11	178.1 (4)
C4—C5—C7—N1	154.4 (3)	N1—C11—C18—O5	−3.3 (6)
C6—C5—C7—C8	−151.8 (3)	C12—C11—C18—O5	−122.7 (5)
C4—C5—C7—C8	31.7 (4)	C10—C11—C18—O5	114.1 (5)
N1—C7—C8—C9	171.7 (3)	N1—C11—C18—O4	170.9 (3)
C5—C7—C8—C9	−57.9 (4)	C12—C11—C18—O4	51.5 (4)
N1—C7—C8—C10	46.0 (3)	C10—C11—C18—O4	−71.8 (4)
C5—C7—C8—C10	176.4 (3)	C8—C10—C20—O2	59.3 (6)
C4—O1—C9—C8	−27.3 (4)	C11—C10—C20—O2	−57.1 (6)
C7—C8—C9—O1	55.1 (4)	C8—C10—C20—O3A	−139.9 (8)
C10—C8—C9—O1	171.3 (3)	C11—C10—C20—O3A	103.6 (9)
C7—C8—C10—C20	−150.5 (4)	C8—C10—C20—O3B	−115.1 (7)
C9—C8—C10—C20	88.9 (5)	C11—C10—C20—O3B	128.4 (7)
C7—C8—C10—C11	−26.8 (3)	O2—C20—O3A—C21A	−16.4 (14)
C9—C8—C10—C11	−147.5 (3)	O3B—C20—O3A—C21A	115 (3)
C7—N1—C11—C18	148.3 (3)	C10—C20—O3A—C21A	−178.5 (9)
C7—N1—C11—C12	−93.0 (3)	C20—O3A—C21A—C22A	178.4 (15)
C7—N1—C11—C10	26.7 (3)	O2—C20—O3B—C21B	7.6 (16)
C20—C10—C11—N1	123.6 (4)	O3A—C20—O3B—C21B	−54.6 (19)
C8—C10—C11—N1	0.5 (3)	C10—C20—O3B—C21B	−178.5 (9)
C20—C10—C11—C18	4.8 (4)	C20—O3B—C21B—C22B	74 (2)
C8—C10—C11—C18	−118.2 (3)		

### Hydrogen-bond geometry (Å, °)

**Table:**

*D*—H···*A*	*D*—H	H···*A*	*D*···*A*	*D*—H···*A*
C8—H8···O5^i^	0.98	2.37	3.317 (5)	163 (1)

Symmetry codes: (i) −*x*+1, −*y*, −*z*.
